# Failure Load and Fatigue Behavior of Monolithic and Bi-Layer Zirconia Fixed Dental Prostheses Bonded to One-Piece Zirconia Implants

**DOI:** 10.3390/ma15238465

**Published:** 2022-11-28

**Authors:** Frank A. Spitznagel, Johanna S. Hoppe, Estevam A. Bonfante, Tiago M. B. Campos, Robert Langner, Petra C. Gierthmuehlen

**Affiliations:** 1 Department of Prosthodontics, Medical Faculty and University Hospital Düsseldorf, Heinrich-Heine University, Moorenstraße 5, 40225 Düsseldorf, Germany; 2 Department of Prosthodontics and Periodontology; Bauru School of Dentistry, University of Sao Paulo, Bauru 17012-230, SP, Brazil; 3 Institute of Systems Neuroscience, Medical Faculty, Heinrich-Heine-University, 40225 Düsseldorf, Germany; 4 Institute of Neuroscience and Medicine, Brain and Behavior (INM-7), Research Center Jülich, 52425 Jülich, Germany

**Keywords:** dental implant, zirconium, ceramics, fatigue, aging, dental restoration failure, materials testing, dental stress analysis

## Abstract

No evidence-based prosthetic treatment concept for 3-unit fixed-dental-prostheses (FDPs) on ceramic implants is currently available. Therefore, the aim of this in vitro study was to investigate the failure load and fatigue behavior of monolithic and bi-layer zirconia FDPs supported by one-piece ceramic implants. Eighty 3-unit FDPs supported by 160 zirconia-implants (ceramic.implant; vitaclinical) were divided into 4 groups (*n* = 20 each): Group Z-HT: 3Y-TZP monolithic-zirconia (Vita-YZ-HT); Group Z-ST: 4Y-TZP monolithic-zirconia (Vita-YZ-ST); Group FL: 3Y-TZP zirconia (Vita-YZ-HT) with facial-veneer (Vita-VM9); Group RL (Rapid-layer): PICN “table-top” (Vita-Enamic), 3Y-TZP-framework (Vita-YZ-HT). Half of the test samples (*n* = 10/group) were fatigued in a mouth-motion chewing-simulator (F = 98 N, 1.2 million-cycles) with simultaneous thermocycling (5–55 °C). All specimens (fatigued and non-fatigued) were afterwards exposed to single-load-to-failure-testing (Z010, Zwick). Statistical analysis was performed using ANOVA, Tukey’s post-hoc tests and two-sample *t*-tests (*p* < 0.05, Bonferroni-corrected where appropriate). All specimens withstood fatigue application. While the effect of fatigue was not significant in any group (*p* = 0.714), the choice of material had a significant effect (*p* < 0.001). Material FL recorded the highest failure loads, followed by Z-ST, Z-HT and RL, both with and without fatigue application. Taken together, all tested FDP material combinations survived chewing forces that exceeded physiological levels. Bi-Layer FL and monolithic Z-ST showed the highest resilience and might serve as reliable prosthetic reconstruction concepts for 3-unit FDPs on ceramic implants.

## 1. Introduction

Today, a large variety of dental materials are currently available for reconstructive dentistry and dental implantology. Modern dental ceramics can be subdivided into esthetic glass ceramics, resin-matrix ceramics and high-strength zirconia ceramics [1,2]. 

Dental implants consisting of pure titanium or titanium alloys are considered to be the gold-standard in replacing missing teeth, due to their biocompatibility, fatigue strength and good long-term results [3,4]. However, the grey color of titanium implants can impair esthetics when a thin soft tissue phenotype or a mucosal peri-implant recession is present [5]. 

Lately, ceramic implants have received increasing interest from dentists and patients due to their positive esthetic outcome and tooth-like appearance [6]. Modern ceramic implants are made of either 3 mol% yttria-doped tetragonal zirconia (Y-TZP) or alumina-toughened zirconia (ATZ) with similar hard and soft tissue integration capacities compared to titanium implants [7,8]. Moreover, in both pre-clinical and clinical studies, zirconia implants demonstrated beneficial behavior concerning plaque affinity and inflammatory conditions, such as mucositis and peri-implant infections [9,10,11]. High survival rates of 94.3–98.4% confirm a positive mid-term performance of zirconia implants after 5 years of clinical observation [12,13,14]. Therefore, ceramic implants can serve as a valuable treatment addendum in the armamentarium of dental implantology for single tooth-gaps and edentulous spaces [15]. However, regarding reconstructive treatment concepts for ceramic implants, no clear consensus can be found in the dental literature. 

A recent systematic review reported a cumulative survival rate of 95% after 5 years for all-ceramic single crowns (SCs) and 3-unit fixed dental prostheses (FDPs) supported by ceramic implants [16]. A detailed analysis of technical complications showed that significantly fewer chipping events occurred with monolithic SCs than with veneered SCs. Similarly, significantly more chipping events were observed with bi-layer FDPs than with veneered SCs over time, which is in line with titanium implants [16,17,18]. 

As traditional veneering systems are prone to delamination failures and chip-off fractures, CAD/CAM fabricated monolithic zirconia restorations should be chosen for posterior SCs and FDPs on implants to decrease technical complications [2,16]. Innovative translucent zirconia ceramics (4Y-TZP, 5Y-TZP) with enhanced optical properties were recently developed to meet the esthetic expectations of patients and clinicians from former opaque 3Y-TZP zirconia restorations [2]. The improved translucency of these zirconia ceramics is, however, accompanied by reduced strength and toughness [2,19].

As with glass ceramics, zirconia restorations benefit from adhesive bonding, especially in cases of compromised retention, high shear forces and minimum ceramic thicknesses [20,21]. Prerequisites for the long-term success of zirconia prostheses are proper pretreatment of the adhesive surfaces, application of specific primers and adequate composite cements [20,22,23,24]. A recent systematic review was able to show that high-translucent zirconia restorations can also be pretreated with the same simplified bonding protocol as 3Y-TZP zirconia, consisting of air-particle abrasion, application of an MDP (10-methacryloyloxydecyl dihydrogen phosphate) or phosphate-monomer based primer and cementation with a self- or dual-cure composite cement [22]. 

Currently, two diametrically opposed paths are being pursued to achieve both esthetic and durable, high-strength restorations. On the one hand, multilayer zirconia discs with color and strength gradient technology were launched to merge the positive mechanical and optical properties of different zirconia generations; on the other hand, the combination of different all-ceramic bi-layer materials is used for esthetic restorations [25,26]. This includes a high-strength zirconia framework with all functional parts in the monolithic core material and a facial layering with felspathic porcelain or a combination of different tooth-like materials [27,28]. 

Currently, no robust prosthetic treatment concept exists for prosthetic reconstructions on ceramic implants. Moreover, wear of zirconia-based restorations is another relevant topic that is extensively discussed within the scientific community, especially when both jaws are restored with different prosthetic materials. So far, no study has investigated possible novel treatment concepts to overcome these issues. A recent in vitro study presented a restorative rapid-layer concept with a polymer-infiltrated ceramic network (PICN) material and a 3Y-TZP framework [28,29]. This design performed well for SCs on ceramic implants, but it is currently unclear if this concept is also transferable to FDPs. 

As neither in vitro nor clinical studies have analyzed the performance of monolithic translucent zirconia restorations or the rapid-layer concept of a PICN material with a zirconia framework as FDPs on ceramic implants, this needs to be investigated. Therefore, the aim of this in-vitro study was to assess the thermo-mechanical fatigue behavior and failure modes of monolithic and bi-layer zirconia FDPs supported by one-piece zirconia implants. The tested null hypotheses assumed: There is no difference in (i) failure load and (ii) fatigue behaviour of bi-layer and monolithic implant restorations on zirconia implants. Monolithic 3Y-TZP implant FDPs served as a control. 

## 2. Materials and Methods

**Figure 1:**
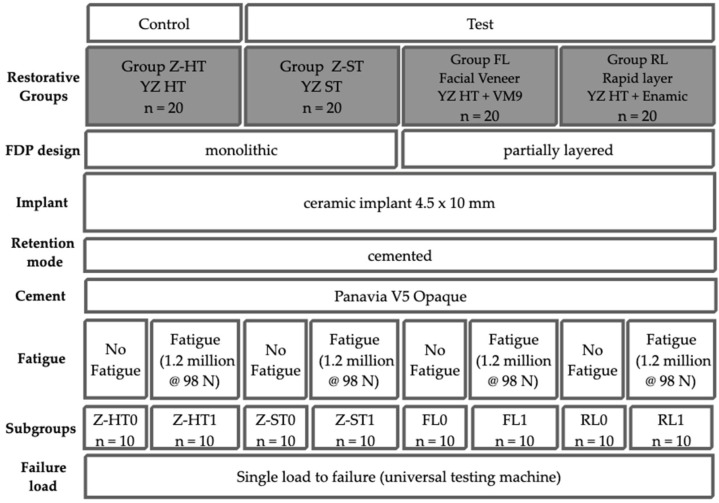
Study set-up.

In total, 80 FDPs with two one-piece zirconia implants (Y-TZP, ceramic.implant 4.5 × 10 mm, vitaclinical, Bad Säckingen, Germany), serving as abutments for a 3-unit FDP, were used. The 80 FDP specimens were split into three test groups and one control group (*n* = 20 per group) depending on the prosthetic material (Figure 1 and Table 1):

- Group Z-HT: monolithic 3Y-TZP zirconia FDP (Vita YZ-HT, Vita Zahnfabrik, Bad Säckingen, Germany)
- Group Z-ST: monolithic 4Y-TZP zirconia FDP (Vita YZ-ST, Vita Zahnfabrik)
- Group FL: 3Y-TZP zirconia FDP (Vita YZ-HT, Vita Zahnfabrik) with facial veneer (Feldspathic porcelain: Vita VM9, Vita Zahnfabrik)
- Group RL: polymer-infiltrated ceramic network (PICN Vita Enamic, Vita Zahnfabrik) “tabletop” resin bonded to a 3Y-TZP zirconia framework (Vita YZ-HT, Vita Zahnfabrik)

### 2.1. Fabrication of Implant Crowns

**Figure 2:**
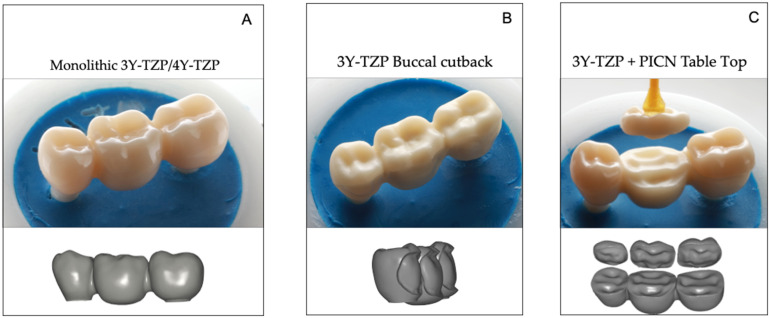
Digital restoration design: (**A**) Master design for monolithic Groups Z-HT and Z-ST; (**B**) Bi-Layer Group FL with facial veneering; (**C**) Split design of Group RL into 3-YTZP framework and PICN tabletop.

To standardize test specimens, all zirconia implants were embedded either in the position of a mandibular second premolar or a second molar in a prosthetically optimal position for a 3-unit fixed-dental prosthesis in a master model (frasaco-Model, frasaco, Tettnang, Germany). An optical impression was made (CEREC Primescan, Dentsply Sirona, Charlotte, USA) and a master design of a 3-unit FDP (InLab 20.0, Dentsply Sirona) was used for all monolithic FDPs (Group Z-HT and Z-ST) to ensure identical and comparable test samples (Figure 2A). Group FL received a facial cut-back design with all functional parts in the monolithic core material (Figure 2B). To guarantee a uniform veneering thickness, silicone keys of the monolithic master designs were produced. Afterwards, all FDPs of Group FL were manually veneered with feldspathic porcelain (VM9, Vita Zahnfabrik). 

For Group RL, a split design from the original master design was used to generate a separate framework and veneer layer (Figure 2C). All implant FDPs were produced by the same master dental technician strictly according to the manufacturer’s recommendation. The connectors of the FDP had an oval shape with a size of 25 mm^2^ (mesial) and 35 mm^2^ (distal). The zirconia FDPs were milled in a five-axis milling unit (Ceramill Motion 2, Amann Girrbach, Pforzheim, Germany). Polymer-infiltrated ceramic (PICN) Tabletops of Group RL were milled out of industrially prefabricated CAD/CAM blocks (VITA Enamic, Vita Zahnfabrik) in a three-axis milling machine (CEREC Primemill, Dentsply Sirona).

### 2.2. Preparation of Specimens

All zirconia implants were perpendicularly embedded in epoxy resin (RenCast^®^ CW20/ Ren^®^ HY 49, Huntsman Advanced Materials, Salt Lake City, UT, USA) [30,31]. Clinically marginal bone losses of 0.7–0.79 mm after 1 year [15,32] and 0.7 ± 0.6 mm after 5 years are reported in the dental literature, therefore, a clearance of 0.5–1 mm between the implant shoulder and resin surface was chosen [12].

### 2.3. Adhesive Cementation

The intaglio surface of the zirconia FDPs of all groups were air-particle abraded with 50 μm aluminum-oxide at a pressure of 2 bar prior to adhesive bonding. Prosthetic restorations were subsequently cleaned with 70% ethanol for 3 min in an ultrasound bath (Bandelin Sonorex, Bandelin, Berlin, Germany), followed by the application of an MDP-containing primer (Clearfil Ceramic Primer Plus, Kuraray Noritake). 

Polymer-infiltrated ceramic “tabletops” of Group RL were etched with 5% hydrofluoric acid (Vita Ceramics Etch, Vita Zahnfabrik) for 60 s, cleaned with water and dried with oil-free air and then a primer (Clearfil Ceramic Primer Plus) was applied. PICN “tabletops” were then adhesively bonded with a self-curing composite cement (Panavia V5 opaque, Kuraray Noritake) to the air-particle abraded and MDP pretreated occlusal surface of the corresponding 3Y-TZP zirconia framework. The MDP Primer (Clearfil Ceramic Primer) was also applied to the zirconia implants. 

Afterwards, all zirconia FDPs were bonded with a self-curing composite (Panavia V5 opaque) to the one-piece ceramic implants. To allow a complete polymerization of the adhesive interface, all specimens were put in distilled water at 37 °C in an incubator (Universalschrank UF 55, Memmert, Schwabach, Germany) for 24 h [33]. Table 2 summarizes the pretreatments and resin cementation of all tested materials.

### 2.4. Fatigue Analysis

Ten specimens from each group were subjected to dynamic thermomechanical loading (5 °C to 55 °C, dwell time 120 s) in a mouth motion fatigue simulator (CS-4.8 professional line, SD Mechatronik, Feldkirchen-Westerham, Germany). An occlusal load of 98 N at 1.2 million chewing cycles with a frequency of 1.6 Hz was applied to the central fossa of the pontic of the restoration, equivalent to a 5-year clinical service time [34,35,36]. A steatite indenter (r = 3 mm, Hoechst CeramTec, Wunsiedel, Germany) was sliding 0.5 mm down the central fossa towards the mesio-buccal cusp, with a vertical movement of 2 mm to simulate aspects of natural chewing. During thermomechanical loading, specimens were investigated regularly for cracks and/or fracture failures as well as for mobility of the restorative suprastructure. 

### 2.5. Single Load to Failure (SLF)

All samples (loaded and non-loaded) were vertically loaded until failure in a universal testing machine (Zwick Z010/TN2S, Zwick Roell, Ulm, Germany). Failure load was applied at the same contact point as during the chewing simulation with a crosshead speed of 1.5 mm/min. A steel ball with a diameter of 6 mm served as a load indenter. Fractures of the ceramic veneer (chipping, cracks) and catastrophic core fractures of the framework/monolithic FDP restoration were defined as failure.

### 2.6. Fractographic Analysis

Firstly, test specimens were investigated under a polarized light microscope (AxioZoom V.16, Carl Zeiss Microscopy, Oberkochen, Germany) after fatigue and SLF. Most meaningful samples were then analyzed for qualitative fractographic evaluation with a scanning electron microscope (Vega 3, Tescan, Kohoutovice, Czech Republic) to determine the failure mode. 

### 2.7. Statistical Analysis

A power calculation (G*Power 3.1.9.7, Düsseldorf, Germany) with the factors (i) type of material (Z-HT, Z-ST, FL, RL) and (ii) fatigue application (yes/no) of bi-layer and monolithic FDPs was performed with respect to statistical testing via ANOVA. A sample size of *n* = 10 per group (*n* = 80 in total) was found to enable detection of any effect of at least large size (Cohen’s effect size of f = 0.38) with 80% power and a two-sided type-I error of *p* < 0.05 for the two factors and their interaction. 

The statistical software SPSS 28 (IBM Corp., Armonk, NY, USA) was used to perform the analysis. To test for homogeneity of variance before using ANOVA, the Levene Test was applied to test for the main effects and interactions of the two factors of interest (type of prosthetic material and fatigue), followed by Tukey’s post-hoc tests for the pairwise comparison of material types. Two-sample *t*-tests were used to test separately for influence of fatigue for each type of material. The *p*-value was defined at <0.05 (95% CI) for all tests, Bonferroni-corrected for multiple comparisons where applicable. Data were graphically displayed in boxplots. 

## 3. Results

### 3.1. Cyclic Loading 

**Figure 3:**
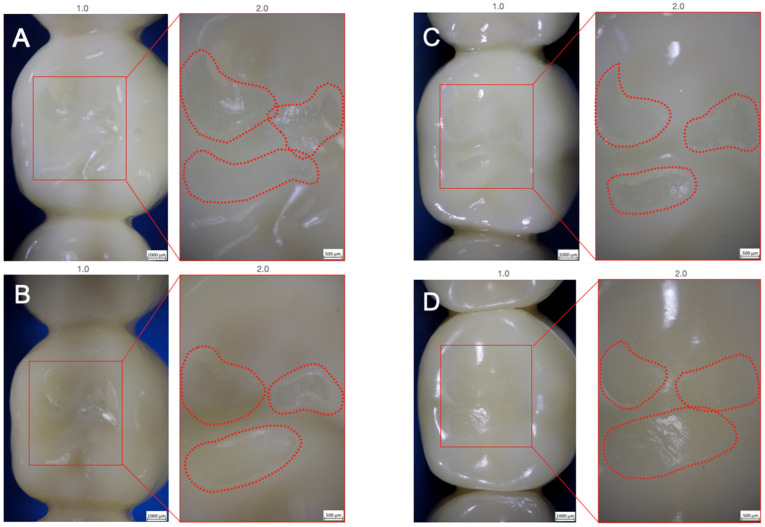
Superficial wear of glazing material for Groups Z-HT (**A**), Z-ST (**B**) and FL (**C**). Clear wear facets of Group RL (**D**) after cyclic loading.

All investigated samples survived thermomechanical loading, leading to a 100% 5-year simulated survival rate. No bulk or cohesive fractures within the FDPs or implants were noticed during and after chewing simulation. Only superficial wear of the glazing material could be observed for Groups Z-HT, Z-ST and FL (Figure 3A–C). Group RL showed wear facets with abrasion of the PICN ceramic on the loading area of the central fossa (Figure 3D). 

### 3.2. Single Load to Failure

**Figure 4:**
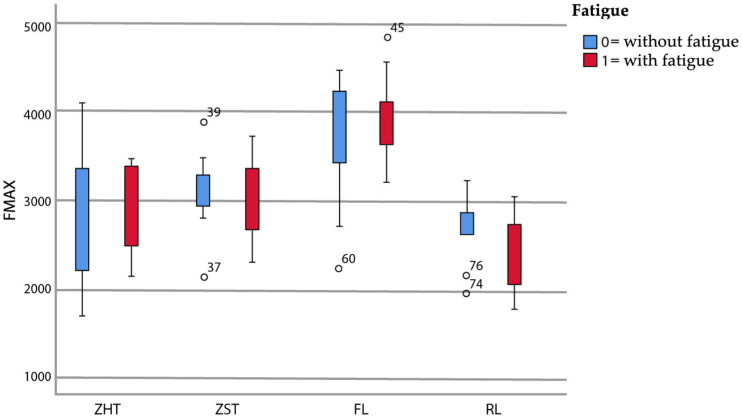
Boxplot of failure load values (in N).

Failure loads after static loading are summarized in Table 3 and graphically displayed in Figure 4.

There was neither a significant main effect of fatigue application, F(1,72) = 0.14, *p* = 0.714, nor a significant interaction between fatigue application and restoration material, F(3,72) = 0.85, *p* = 0.469. However, the main effect of restoration material was significant, F(3,72) = 19.20, *p* < 0.001. *t*-tests for the pairwise comparison between specimens with and without fatigue exposure for each material type confirmed this and showed no significance (Table 3). 

The highest failure loads were observed for material FL both without prior fatigue application (FL0: 3662 N; FL0 > Z-ST0 > Z-HT0 > RL0) and with prior fatigue application (FL1: 3923 N; FL1 > Z-ST1 > Z-HT1 > RL1). Group RL showed the lowest failure loads of all materials tested, both without and with fatigue application. 

Irrespective of fatigue, material FL showed significantly higher failure loads relative to all other three materials tested (*p* < 0.05), except for the comparison of FL0 and Z-ST0 (*p* = 0.357) (Table 3). No difference could be detected between monolithic 3Y-TZP Z-HT and 4Y-TZP Z-ST (*p* = 1.000) and between Z-HT and RL (*p* ≥ 0.197), regardless of fatigue application (Table 3). 

Materials Z-ST and RL showed a statistical difference in failure load with fatigue application (*p* = 0.044) (Table 3) but not without fatigue (*p* = 0.482). 

### 3.3. Failure and Fractographic Analysis after Single Load to Failure Testing

**Figure 5:**
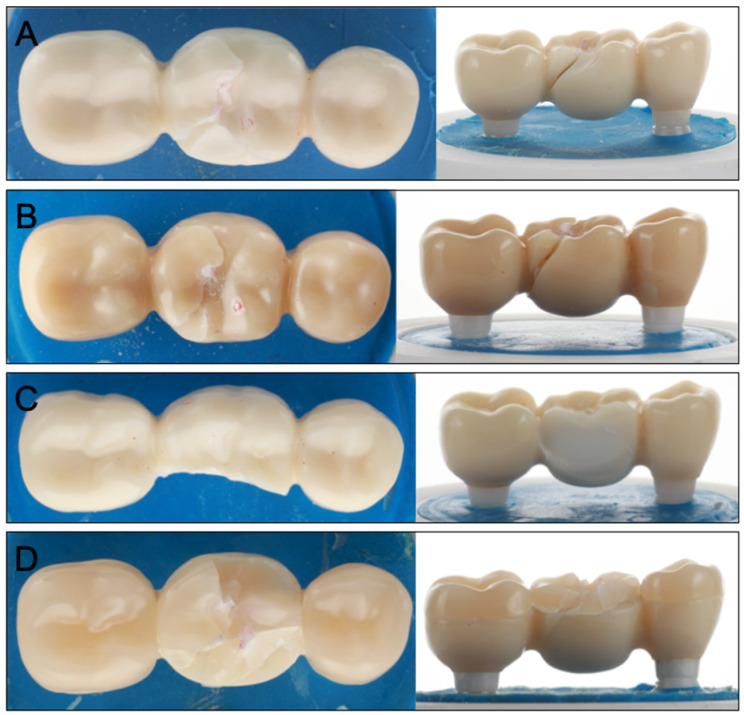
Representative samples of all Groups after single-load-to-fracture assessment. Light microscopy shows (**A**) occlusal and side view of Group Z-HT, bulk fracture of FDP within the distal connector (**B**) Group Z-ST, bulk fracture of FDP within the distal connector (**C**) Group FL, bulk fracture without involvement of the distal connector (**D**) Group RL, chipping fracture of PICN and bulk fracture of the zirconia framework.

**Figure 6:**
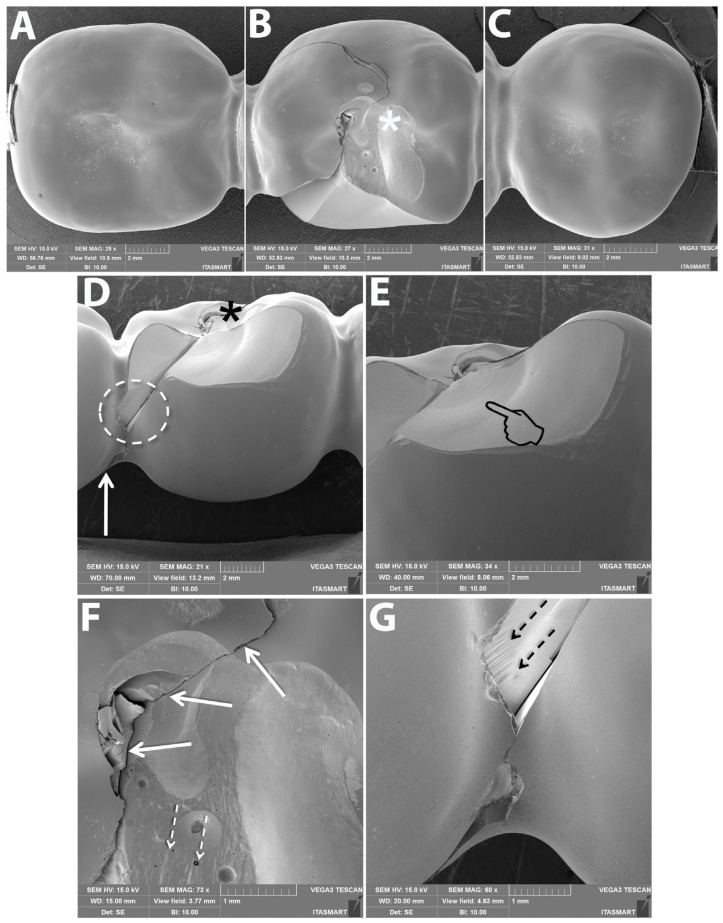
Representative scanning electron microscopy (SEM) micrographs of fractured monolithic FDP from Group Z-HT. Occlusal overviews of abutments (**A**,**C**) show intact marginal ridges and connectors whereas the pontic (**B**) presents a cohesive fracture at the occlusal (asterisk) and buccal surfaces, as well as a crack that extends towards the distal abutment. (**D**) Buccal overview shows loading area at the occlusal surface where the fracture seems to have originated (asterisk) and a crack that extends down to the gingival part of the connector. In a magnification view of the cohesive surface fracture at the buccal surface (**E**) arrest lines (pointer) with their concave portion pointing towards the occlusal surface indicate the fracture origin. (**F**) is a magnification of the occlusal surface where the solid arrows show a crack initiating at the occlusal and propagating down towards the gingival portion of the connector and the other crack front showing its direction towards the buccal surface, eventually leading to the cohesive fracture at the buccal surface (suggested by the hackle lines pointed by the dotted arrows). (**G**) is a magnification of the area circled in D where the main buccal cohesive fracture ended at the mesial portion of the FDP distal abutment showing hackle lines (dotted arrows) indicating the direction of crack propagation.

**Figure 7:**
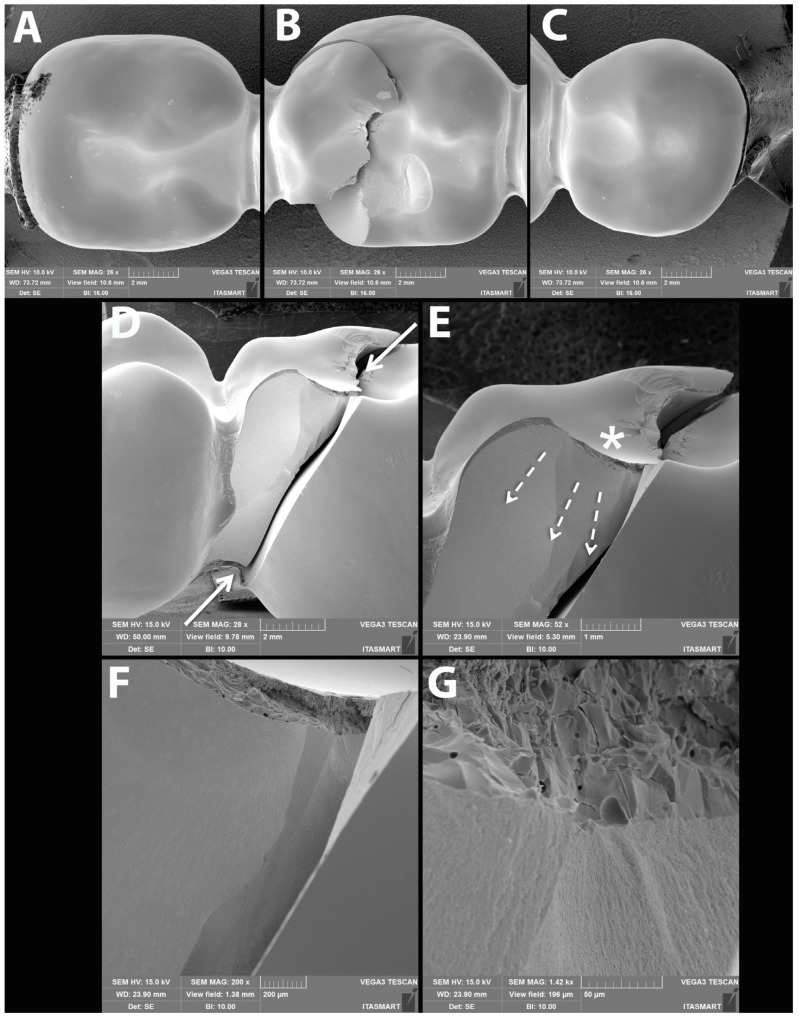
Meaningful specimen of Group Z-ST after single-load-to-failure assessment. (**A**–**C**) are an overview of the occlusal surfaces of an entire monolithic Z-ST FDP showing intact abutments (**A**,**C**) and the fractured pontic (**B**). The buccal view in (**D**) shows a crack that connects the occlusal surface to the gingival portion of the connector of the pontic. Magnifications of the buccal surface are shown in the subsequent images where the asterisk indicates the possible fracture origin site and the dotted arrows the direction of crack propagation (**E**). (**F**,**G**) are magnifications of the damage caused by the indenter at the occlusal surface.

**Figure 8:**
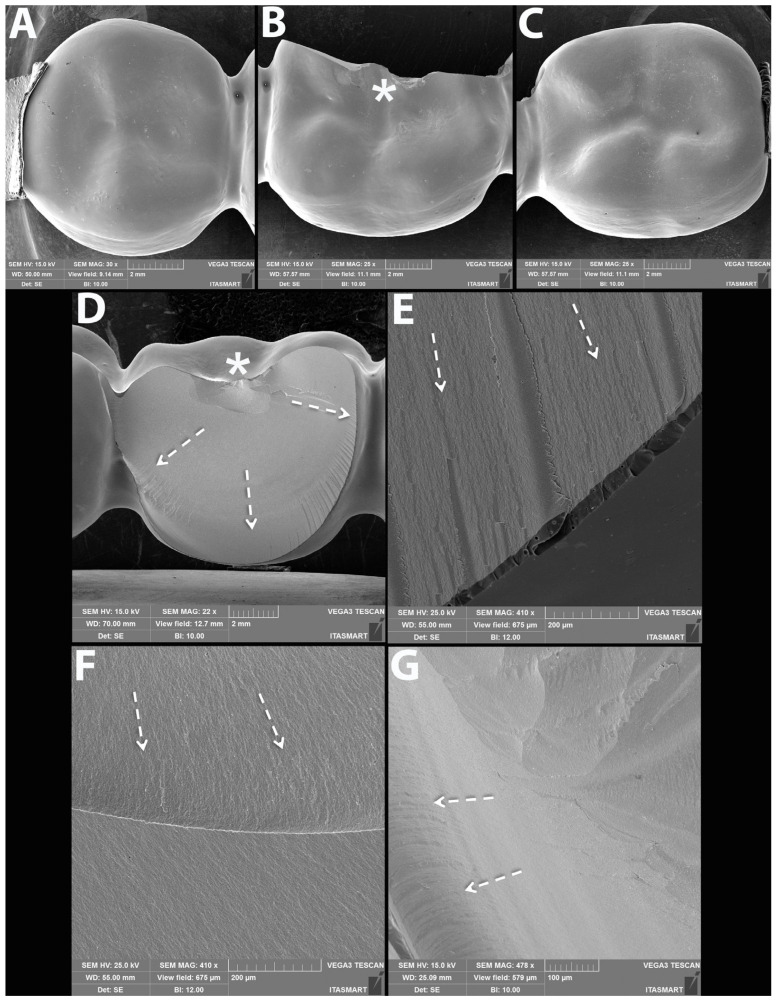
SEM micrographs of fractured FDP from Group FL. Occlusal views of abutments (**A**,**C**) and pontic (**B**) of an FDP showing the fracture initiation site at the asterisk. (**D**) is a buccal overview of the fractured surface of the pontic showing the origin (asterisk) and direction of crack propagation (dotted arrows). Clockwise magnification of (**D**) is presented in subsequent micrographs (**E**–**G**) where dotted arrows point at the hackle lines indicating the direction of crack propagation from the occlusal surface towards the margins.

**Figure 9:**
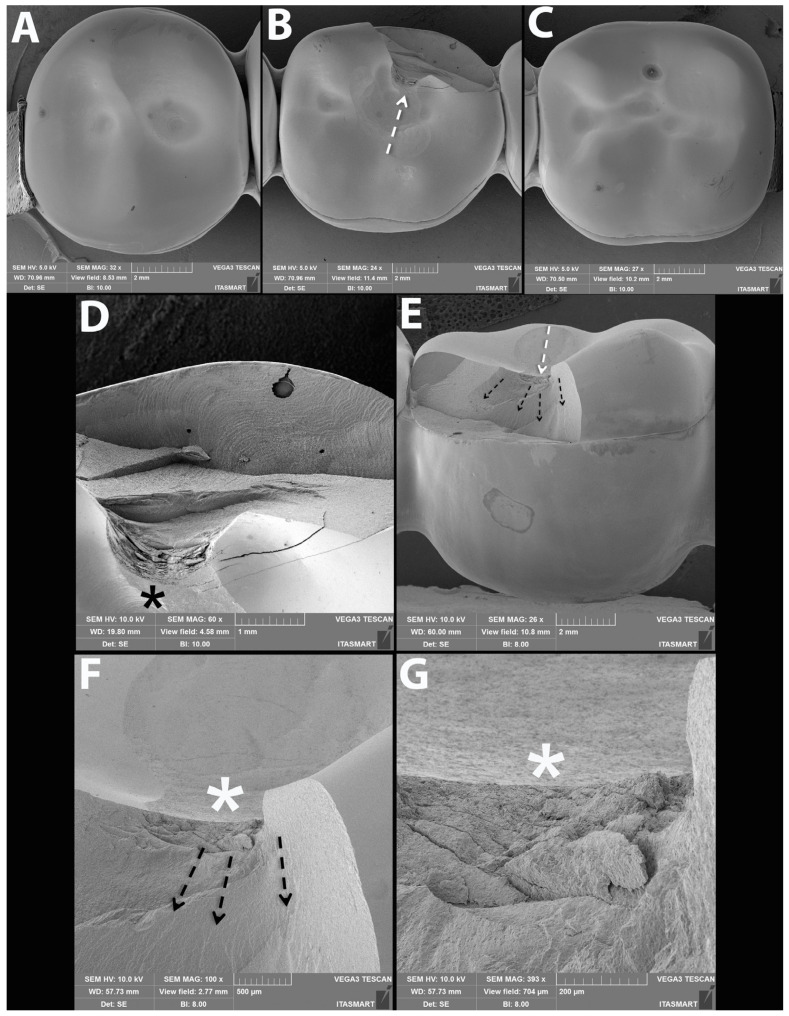
Representative SEM micrographs of fractured FDP from Group RL. Occlusal views of abutments (**A**,**C**) and of fatigued pontic (**B**) with indenter sliding path (dotted white arrow) leading to discrete wear of the PICN tabletop bonded to the zirconia framework. (**D**) Magnified view of the fracture origin at the occlusal surface (asterisk) and (**E**) buccal view showing the origin (dotted white arrow) and direction of crack propagation (dotted black arrows) within the PICN material halted at the interface with the zirconia framework. (**F**,**G**) are magnified views of E where the dotted arrows of (**F**) point at the hackle lines indicating the direction of crack propagation towards the zirconia framework and (**G**) shows damage from loading at the occlusal region (asterisk).

All FDPs of Z-HT, Z-ST and FL1 revealed bulk fractures within the distal connector after single load to failure (Figure 5 A,B and Table 4). FDPs of Group FL0 predominately suffered also from bulk fractures within the distal connector (Table 4). Bulk fracture without involvement of the connector was observed in two samples (20%) of material FL0 (Figure 5C and Table 4). Material RL predominately showed fractures within the PICN Enamic Tabletop (80%), whereas the underlying FDP framework revealed only fractures in a few specimens (20%) (Figure 5D and Table 4). Detailed fractography analysis of representative specimens of each Group is given in Figure 6, Figure 7, Figure 8 and Figure 9.

## 4. Discussion

Zirconia ceramics and their different generations with increased translucency and beneficial esthetics, as well as the combination into strength-gradient multilayer pucks, are extensively investigated in current scientific research. In addition to zirconia, resin-matrix ceramics, such as PICN and CAD/CAM composites are widely analyzed due to their attractive application for chairside workflow and efficient machinability and processing [37]. Moreover, additive CAD/CAM methods are of interest in reconstructive dentistry and presently exploited [38]. 

This laboratory study investigated the failure load and fatigue behavior of different designs of monolithic translucent zirconia, 3Y-TZP with a facial veneer and rapid-layer posterior FDPs bonded to one-piece zirconia implants. The tested null hypothesis was partially rejected as the type of restoration material affected the failure load. 

All tested materials survived dynamic loading up to 1.2 million cycles with simultaneous thermocycling, resulting in a 100% in vitro survival rate after chewing simulation. Measured failure loads (>2384 N) of all implant-supported FDPs surpassed normal physiological occlusal forces of 200–900 N in the posterior dentition, irrespective of fatigue application [39]. Accordingly, this may suggest that all tested restorative concepts are suitable for clinical use as 3-unit FDPs on one-piece zirconia implants in the posterior region. 

To the authors’ best knowledge, this is the first in vitro study that investigated different restorative concepts for FDPs on ceramic implants. Furthermore, no laboratory studies with monolithic all-ceramic FDPs on titanium implants are reported in the dental literature. Therefore, a comparison to other studies is difficult. 

Most in vitro studies investigated different posterior bi-layer 3Y-TZP FDPs on titanium implants [40,41,42,43,44,45]. Studies without thermodynamical loading and solely water storage for up to 48h recorded failure loads of 692 N (30° angulated) [40], 2086 N (axial) [41] and 1380–2690 N (axial) [43]. Failure loads of 2465–2583 N (600,000 cycles, 50 N) [44] and 2086 N (100,000 cycles, 20–200 N) [45] were reported for bi-layer 3Y-TZP FDPs with fatigue application. A study with a similar test design (1.2 m. cycles, 100 N), as in the present study, measured failure loads of 1636N in an axial loading setting and 1086 N with angulated loading forces [42]. All reported failure loads are in the range or below the lowest recorded values (<2384 N) of the present study. 

The highest failure loads both without and with fatigue application were measured for Group FL. Different from the monolithic groups Z-HT and Z-ST, group FL had a porcelain veneer over a 3Y-TZP framework. It is possible that t-m phase transformation due to moisture of the porcelain slurry may have led to compressive stresses, eventually increasing the load to failure [46]. Another possible explanation might be an improved hardness, density and surface without irregularities and defects due to the increasing number of firings during the veneering process [47,48]. Moreover, the different restorative designs might have an impact on force absorption and stress distribution, ultimately leading to different failure loads. 

No differences between failure loads of monolithic 3Y-TZP and 4Y-TZP FDPs could be detected. This is in line with a previous study in which no statistically differences could be found between failure loads of the same 3Y-TZP and 4Y-TZP material on SCs [28]. Therefore, monolithic 4Y-TZP might be an esthetic and durable alternative for 3-unit FDPs on one-piece ceramic implants. Clinical studies reporting on 3Y-TZP monolithic zirconia FDPs on titanium implants detected high survival rates of 100% after one and 96% after 3 years [49,50]. These high short-term survival rates confirm that monolithic reconstructions might be preferred not only for titanium but also for ceramic implants [16,51]. 

The lowest failure load values were measured for Group RL. As this might be expected with a PICN material as a tabletop on a 3Y-TZP framework, loads were nevertheless high. Most of the observed fractures would be repairable in a clinical scenario with either a new chairside fabricated PICN tabletop or a direct repair with a resin composite. 

The PICN material, with mechanical properties imitating a human tooth, such as a similar wear behavior and a dampening effect, might protect the implant from uncontrolled high occlusal forces and overloading [52]. The results of this study suggest that the tested rapid-layer material combination proved to be an attractive material combination not only for single-crowns, but also for 3-unit FDPs [28]. The easy replaceability of the PICN material in the event of fractures or extreme wear, can serve as a cost- and time-effective restoration concept. 

Failure and fractographic analysis of monolithic zirconia FDPs resulted in 100% bulk fractures with and without fatigue application. The fracture originated from the occlusal surface in the zone of the highest compressive forces down to the distal connector of the 3-unit FDP. As the contact area was on the pontic site and not along the axis of the implants, this unfavorable loading condition resulted in the gingival portion of the connector becoming the weakest link due to localized tensile stress with the highest probability of fracture and also an origin of a competing failure mode [53]. 

For Group FL, almost all of the specimens showed the same fracture pattern with bulk fractures through the distal connector, except two specimens which showed bulk fractures through the pontic. Interestingly, the facial veneering withstood the high forces and did not fail before bulk fracture. 

However, Group RL showed a different failure pattern: 80% of the specimens showed chipping fractures of the PICN tabletops without detachment and involvement of the 3Y-TZP substructure. Only 20% of the RL specimens fractured through the distal connector as the other groups. 

According to the present findings all tested monolithic and bi-layer all-ceramic restorations can serve as 3-unit FDPs on one-piece ceramic implants, whereas facial veneered 3Y-TZP and monolithic translucent 4Y-TZP FDPs presented the highest load to failure values. 

A limitation of this study is that the obtained result of the implant-restoration complex might be only transferred to a certain limit to other available one-piece ceramic implants. Future studies should compare the proposed restorative rapid-layer concept with novel strength-gradient multilayer zirconia SCs and FDPs. Other materials, such as bioactive restoratives, should also be explored as potential prosthetic materials [54,55]. Likewise, recently introduced two-piece ceramic implants with a screw-connection should be investigated in comparison to one-piece ceramic implants, as they allow increased prosthetic flexibility and retrievability of the suprastructure. 

## 5. Conclusions

The following conclusions can be drawn based on the obtained results and within the limits of this laboratory study:

1. The applied fatigue protocol had no effect on the failure load of the materials investigated.
2. All tested prosthetic reconstructions showed higher failure loads (>2384 N) than normal physiological occlusal forces (200–900 N) in the posterior region and can be used clinically.
3. Bi-Layer FL and monolithic Z-ST showed the highest resilience and might serve as reliable prosthetic reconstruction concepts for 3-unit FDPs on ceramic implants.
4. The Rapid-Layer concept shifted failure modes from catastrophic bulk fractures to clinically repairable failures within the PICN veneer layer.
5. The proposed rapid-layer design with a PICN “tabletop” might be an interesting restorative treatment concept for zirconia implants due to their tooth-like wear behavior and easy replaceability.
6. Proper veneer design with a limited extension to the buccal/facial area resulted in superior failure load results.
7. Future research should focus on novel strength-gradient multilayer zirconia ceramics for SCs and FDPs on one- and two-piece ceramic implants.

## Figures and Tables

**Table 1:** Prosthetic materials with group names and studied implant with characteristic flexural strength according to manufacturer’s information.

Group (*n* = 20)	FDP /Implant Design	Type	Name	Y_2_O_3_ [Weight%]	Flexural Strength [MPa]	Manufacturer
Z-HT	Monolithic FDP	3Y-TZP Zirconia	Vita YZ HT	4–6	1200	Vita Zahnfabrik, Bad Säckingen, Germany
Z-ST	Monolithic FDP	4Y-TZP Zirconia	Vita YZ ST	6–8	>850	Vita Zahnfabrik, Bad Säckingen, Germany
FL	Bi-layer FDP	Feldspathic Veneer	VM9	-	100	Vita Zahnfabrik, Bad Säckingen, Germany
		3Y-TZP Zirconia	Vita YZ HT	4–6	1200	
RL	Rapid- Layer FDP	PICN	Vita Enamic	-	150–160	Vita Zahnfabrik, Bad Säckingen, Germany
		3Y-TZP Zirconia	Vita YZ HT	4–6	1200	
All groups	One-piece Implant	3Y-TZP Zirconia	ceramic. implant	5	1400	vitaclinical, Bad Säckingen, Germany

**Table 2:** Pretreatment and adhesive cementation of tested materials.

	Zirconia FDP’s	Polymer-Infiltrated Ceramic Tabletop	Zirconia Implant
Group	Z-HT, Z-ST, FL, RL (framework)	RL	All
Surface Pre-treatment	Air-particle abrasion with 50 μm Al_2_O_3_ at 2 bar, Ultrasonic cleaning with 70% ethanol for 3 min	Cleaning with 70% Ethanol, Etching with 5% hydrofluoric acid for 60 s (Vita Ceramics Etch, Vita Zahnfabrik), rinsed with air-water spray (30 s), ultrasonic cleaning with distilled water (5 min)	-
Primer	Clearfil Ceramic Primer Plus (Kuraray Noritake)		
Resin Cement	Panavia V5 opaque (Kuraray Noritake)		

**Table 3:** Descriptive statistics of failure load in [N]. Different superscript letters per column without (small letters) or with fatigue exposure (capital letters) indicate statistically significant differences (*p* < 0.05) between materials. Results of *t*-tests (*t*-value, *p*-value) show the effect of fatigue for each type of material (per row).

Group	Without Fatigue	With Fatigue	Influence of Fatigue	
	Mean ± SD	Mean ± SD	*t*-Value	*p*-Value
Z-HT	2899 ± 754 ^b^	2866 ± 509 ^B,C^	0.115	0.910
Z-ST	3138 ± 463 ^a,b^	3000 ± 460 ^B^	0.668	0.512
FL	3662 ± 723 ^a^	3923 ± 527 ^A^	−0.922	0.369
RL	2654 ± 374 ^b^	2384 ± 438 ^C^	1.481	0.156

**Table 4:** Failure analysis of FDPs after SLF. Chipping of Group RL is referred to the PICN Tabletop.

Group	Z-HT		Z-ST		FL		RL	
	Z-HT0	Z-HT1	Z-ST0	Z-ST1	FL0	FL1	RL0	RL1
Bulk fracture within connector	10/10 (100%)	10/10 (100%)	10/10 (100%)	10/10 (100%)	8/10 (80%)	10/10 (100%)	3/10 (30%)	1/10 (10%)
Bulk fracture without connector	-	-	-	-	2/10 (20%)	-	-	-
Chipping	-	-	-	-	-	-	7/10 (70%)	9/10 (90%)

## Data Availability

The data presented in this study are available on request from the corresponding author.
